# Key Areas of Preventive Home Visits and Their Importance for Older Adults: A Scoping Review

**DOI:** 10.1111/scs.70133

**Published:** 2025-10-08

**Authors:** Anna Nivestam, Julia Jansson, Josefine Jöninger, Cecilia Pettersson

**Affiliations:** ^1^ Faculty of Health Sciences, Department of Nursing and Health Sciences Kristianstad University Kristianstad Sweden

**Keywords:** autonomy, community care, health promotion, health service, house calls, literature review, nurse, nursing, older people, prevention

## Abstract

**Background:**

An updated review of preventive home visits (PHVs) to older adults is needed to guide future interventions. Nurses play a crucial role in PHVs, and the key areas during this type of visit are important to describe. Moreover, the perspectives of older adults on the visits would provide a foundation for future guidelines about PHVs. Such guidelines could promote equal health conditions. The aim of this scoping review was to identify and synthesise research on key areas in PHVs for older adults, as well as visualise older adults' perspectives.

**Method:**

This is a scoping review inspired by the Joanna Briggs Institute guidelines for scoping reviews.

**Findings:**

Four key areas of nursing practice in PHVs were identified in this scoping review: Supporting older adults' autonomy and independence, Creating a relationship between the older adult and the nurse, Advising to prevent ill health and promote health, and Providing helpful information. Moreover, the following categories described older adults' perspective: Ranging from improving well‐being to offering nothing of benefit, Maintaining independence and autonomy, Having a person to contact, and Appreciating information, advice, and support.

**Study Limitation:**

PHVs are not always conducted by nurses, and some of the included articles involve multiple professions in the visits. This makes it difficult to argue that the findings represent only visits conducted by nurses.

**Conclusion:**

PHVs for older adults can help those adults maintain independence and autonomy in older age. Having a contact person seems to be important not only when it comes to illness but also for maintaining or improving health and well‐being.

## 1 Background

For older adults to live well and remain in their ordinary housing, health‐promoting and preventive efforts may be necessary. Health promotion is a process aimed at maintaining or improving health by increasing control over factors that influence health [1]. On the other hand, preventive work involves identifying risks and problems to avoid ill health [2]. Nurses play a crucial role in the health‐promoting and preventive efforts directed towards older adults. It is often the nurse who coordinates the health care provided in the home environment [3].

One way to promote health [1] and prevent ill health [2] is to offer PHVs. The purpose of PHVs is to help older adults enhance their control over their health, support their functional ability, and reduce the risk of illness by encouraging behaviour modification [4]. Previous studies indicate that PHVs are usually conducted by a nurse [4]. A recently published systematic review shows that complex health interventions that involve a medication review, advice about nutrition and exercise, and which are usually included in PHVs, could increase the odds for a person to live at home [5]. The same patterns could be seen in a systematic review focusing explicitly on PHVs, showing that functional decline could be reduced by an assessment of multiple factors and follow‐up [6]. Moreover, a study that models the cost‐effectiveness of complex health interventions such as PHVs suggests that these visits can be cost‐effective [7]. However, comparing research and drawing valid conclusions is challenging because the content of these visits is very heterogeneous. There are differences in who is offered visits, the aim of the visits, and the topics covered during the visits [8]. There are systematic literature reviews that have described the content of the visits [4, 9], but these reviews are relatively old, and new research has emerged in this field. An ongoing systematic review will investigate the visits' effect on quality of life [10] and a recent systematic review investigated resilience, health, psychology, and social outcomes [11]. However, these reviews do not capture key aspects of nursing practice in PHVs or the perspectives of older adults.

Since nurses seem to be the main professionals who conduct health visits around the world [4] it is important to describe the key areas of nursing practice in PHVs. In addition, there is a lack of a review of the latest research reflecting older adults' perspectives on PHVs. Visualising older adults' perspectives is important to make sure that the visit is targeting their needs. Therefore, an updated review of the visits is needed to guide future interventions. A compilation of the key areas of nursing practice in PHVs and the perspectives of older adults on the visits would provide a foundation for future guidelines about PHVs. Such guidelines could promote equal health conditions and set the standards for future practices.

This scoping review aimed to identify and synthesise research on key areas in PHVs for older adults, as well as visualising older adults' perspectives.

## 2 Methods

### 2.1 Design

This scoping review was inspired by the Joanna Briggs Institute (JBI) guidelines for scoping reviews, which provide a rigorous and transparent framework for identifying, selecting, and synthesising relevant literature on a broad topic [12]. Moreover, PRISMA‐ScR (Preferred Reporting Items for Systematic Reviews and Meta‐Analysis extension for Scoping Reviews) was also used to guide the process [13].

### 2.2 Research Questions of the Literature Analysis

– What are the key areas of nursing practice in PHVs?
– What are the older adults' perspectives to PHVs?

### 2.3 Inclusion and Exclusion Criteria

Empirical studies on PHVs from various parts of the world were identified through searches in eligible databases. For inclusion and exclusion criteria see Table 1.

**TABLE 1:** Inclusion and exclusion criteria of the literature search.

Criteria	Inclusion criteria	Exclusion criteria
Language	English or Swedish	Sources of evidence that were published in languages other than English and Swedish
Year of publication	2013–2023	
Target group	Older adults aged 65 years and older living in ordinary housing	Populations other than older adults were excluded
Intervention	Scheduled visits by a health professional (in this case a nurse) to an older person's home to promote health and prevent ill health	Sources of evidence focused on home visits for specific health conditions, such as diabetes, hypertension, or dementia
Person offering the PHVs	Nurses, district nurses, registered nurses	Visits were nurses not included as part of the intervention

### 2.4 Search Strategy

The search strategy was developed and conducted during the autumn of 2023 by two nurses training to become district nurses (the second and third authors), who were well experienced in providing nursing care to older adults. But they have limited experience in developing and conducting literature research and analysing the literature. Therefore, all searches were made in consultation with the fourth author and an experienced librarian. The search strategy aimed to identify all relevant sources of evidence published in English or Swedish from January 2013 to December 2023, to capture the most recent and updated scientific literature on PHVs, to be able to guide future interventions. The following electronic databases were searched: PubMed and CINAHL Complete. The search terms were derived from the population, concept, and context of interest, and were combined using Boolean operators and truncation symbols. Search terms were narrowed according to the inclusion and exclusion criteria. The search terms included: Aged, Nurse/specialist nurse, Preventive health services, and Home visit. Eligible synonyms for all terms were used in both databases. The reference lists of the included sources of evidence were also screened for additional relevant literature.

### 2.5 Data Selection

The second and third authors separately screened the titles and abstracts of the articles found in the databases, including articles using predefined inclusion and exclusion criteria. Thereafter, full‐text articles were read by the second and third authors to ensure that they met the purpose of the review. Continuously during this process, the second and third authors had supervision from the fourth author. This was due to the second and third authors having limited experience in conducting literature reviews. The fourth author has experience conducting scoping reviews. In addition, reference lists were skimmed for additional articles to include. Discrepancies between the reviewers were resolved by discussion or consultation with the fourth author. The full texts of articles found in the reference lists were retrieved and further assessed for inclusion by the second and third authors, using the same criteria.

### 2.6 Data Extraction

A data extraction table was developed based on the research question. The data extraction table consisted of the following items: author, year, setting, aim, title, study population, research design, methods, description of PHVs, and results based on research questions. The second and third authors separately extracted the data from the included sources of evidence, using the data extraction table. Any disagreements or uncertainties were discussed and resolved by consensus or by involving the fourth author.

### 2.7 Data Analysis

The second and third authors synthesized data according to the research questions [12]. Findings addressing the aim of this review were described under their respective research questions. Subsequently, the second and third authors colour‐coded the text to identify categories, thus enabling a descriptive presentation. The findings were presented with main categories based on the review's research questions. The categories identified were presented under each research question as subheadings. The second and third authors were supervised during the whole analysis process by the fourth author. This means that the fourth author did check the entire process. After that, the first and fourth authors validated and optimized the findings. The first author has been a consultant during the whole research process as well as written the final draft for publication. Finally, all authors discussed and agreed upon the final findings.

## 3 Findings

**FIGURE 1:**
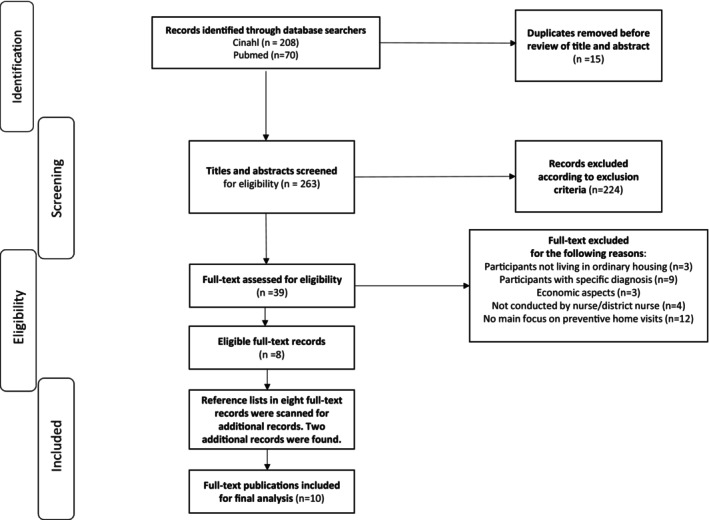
Flow diagram of the phases of the literature search (inspired by [14]).

In total, 278 articles were found, of which 15 were duplicates and therefore removed. Of the 263 remaining articles, 224 were excluded after screening the titles and abstracts. Subsequently, 39 articles were read in full text, and eight articles were found to match the inclusion and exclusion criteria and the aim of the review. In addition, screening the reference lists resulted in two additional articles to include. (Figure 1, flow chart).

The ten included articles were from Europe: Sweden (*n* = 4), Norway (*n* = 4), Germany (*n* = 1), and Netherlands (*n* = 1). An overview of the design of the studies is available in Table 2. Different perspectives such as those of nurses, district nurses, and older adults are represented in the findings. The findings are presented according to our research questions and described under categories (Table 2).

**TABLE 2:** Characteristics of the original scientific publications included in the review (*n* = 10).

First author, year, setting (country)	Title	Aim	Study population	Research design, methods and description of preventive home visits (PHVs)	Main results based on research questions *1. What are the key areas of nursing practice in PHVs?* *2. What are the older adults' perspectives to PHVs?*
Behm et al. 2013 Sweden [15]	Preventive home visits and health – experiences among very old people	To describe the variations in older people's (80+) experiences of a single preventive home visit and its consequences for health	17 participants (80–92 years old)	Qualitative design, semi‐structured interviews analysed using the phenomenographic method No follow‐up A PHV is described as follows: a single home visit made by a nurse, physiotherapist, qualified social worker or occupational therapist. Verbal and written information and advice are given about what the municipality could provide in the form of local meeting places, activities, physical training, walking groups etc. Information about various kinds of help and support, accessibility to assistive devices and housing modifications is provided. Environmental fall risks in the home are identified, and advice on how to prevent them is included. Information is also given about whom the older person could contact about different problems	1: Older adults were given information on how they could avoid accidents during dangerous activities by getting outside help with snow shovelling, changing light bulbs and hanging curtains Teamwork with physical therapists to prevent disability. Older adults received advice about laws, regulations, home care and assistive devices. Nurses could, for example, provide a shower chair. Information and brochures were given in person. The person who completed the PHVs became the contact person. 2: The older adults felt like important members of society through the PHVs. Some felt too healthy to need PHVs and some were too ill which prevented them from participating or they did not want to think about the future. The older adults appreciated nurse contact, which created security. Older adults appreciated talking about risk factors and receiving brochures in person. New knowledge could lead to changed behaviour. Some did not feel that the PHVs provided information they found useful
Blotenberg et al. 2023 Germany [16]	Acceptance of preventive home visits by nurses – The user perspective	To investigate the acceptance of PHVs for older adults in rural Germany	15 participants (65–85 years old)	Qualitative descriptive approach, semi‐structured interviews and content analysis. Follow‐up: 4 PHVs were conducted over 10 months. A PHV is described as follows: during the first home visit, the Standardised Assessment of Elderly People in Primary Care in Europe with the module mobility (STEP‐m) is performed as the basis for individual counselling and referral to support services. The second home visit constitutes the first consultation session, and a prevention plan according to the priorities of the needs specifically chosen by the person affected is handed out. The third and fourth home visits represent the continuation of consultation and the evaluation	1: Older adults received information about various activities. Nurses collaborated with physical therapists. PHVs gave them a fixed nurse contact. A close relationship with the nurse was important, and the fact that the nurse came from the area and spoke the same dialect was significant. 2: PHVs increased the older adults' self‐confidence, and their well‐being increased by feeling seen. The relationship with the nurse was perceived as almost friendly. They appreciated that the counselling was based on individual needs and that the knowledge led to more informed choices and independence was promoted. Through information about activities, the older adults could make new contacts. PHVs encouraged self‐reliance on one's own resources. Having a regular contact nurse was appreciated and contributed to a reduced feeling of helplessness, and uncertainty
Lagerin et al. 2016 Sweden [17]	District nurses' experiences of preventive home visits to 75‐year‐olds in Stockholm: a qualitative study	The first aim of this study was thus to describe the dialogue between DNs and older people in PHVs from the perspective of the DNs. The second was to identify barriers to and facilitators of this dialogue as perceived by the DNs	20 participants (75 years old)	Qualitative descriptive design. Group interviews with DNs are analysed with qualitative content analysis. No follow‐up A PHV is described as follows: DNs offer older adults the opportunity to discuss health and health problems in a structured health dialogue in order to: (1) identify the older people's health concerns related to their living environment, (2) support self‐care activities and empowerment, (3) use a person‐centred salutogenic approach, (4) use and evaluate nursing care interventions and (5) document nursing care interventions in accordance with the Well‐being–Integrity–Prevention–Safety (VIPS) model	1: The older adults received information about fall prevention. The relationship with the older adults was promoted by the fact that the PHVs was carried out in the home environment. 2: PHVs promotes a good relationship and builds trust in the DN. It promotes a holistic assessment of the older adults' health
Nivestam et al. 2021 Sweden [18]	Older person's experiences of benefits gained from the support and advice given during preventive home visits	To explore older person's experiences of the benefits gained from the support and advice given during the PHV	13 participants (76–91 years old)	Qualitative study conducted and analysed using content analysis. No follow‐up A PHV is described as follows: Questions to assess different aspects of health covering areas such as nutrition, physical health, mental health, housing and finances. Advice and support are individually tailored concerning, for example, how to prevent the risk of falling, physical exercise, and information about services the municipality can offer. Various brochures with information such as nutritional advice are provided. Support could be offered for example by talking about loneliness	1: Older adults received a brochure with information about where they could turn if they needed help. They received feedback on their eating habits and physical activity. 2: PHVs contributed to the older adults feeling appreciated. Regular nurse contact and knowing where to turn if needed contributed to a sense of security. That eating habits and physical activity were assessed contributed to feelings of security. Information brochures were appreciated. Information about diet and exercise was appreciated. Conversations about risk factors helped the older adults feel prepared for the future
Sherman et al. 2014 Sweden [19]	Effects of preventive home visits by district nurses on self‐reported health of 75‐year‐olds	To analyse the effects of PHVs by DNs on the self‐reported health of 75‐year‐olds, including changes in self‐reported health after the visits. The study also investigated whether or not the participants believed the visit was useful	Participants 75 years old: study group *n* = 176, control group *n* = 262	A controlled design. A cluster‐controlled Trial. Questionnaires. Follow‐up: DNs and older adults decide whether follow‐up contact is needed. A PHV is described as follows: a health dialogue with assessment of health, planning, diagnosis of health needs, nursing intervention and evaluation of nursing care. Information about activities in the area where they live, and a brochure about safety at home	1: Not described 2: PHVs contributed to feeling valued. The older adults felt safe if they had a good relationship with a sense of trust. A few did not think PHVs led to anything because they felt too healthy. More older adults in the study group who received PHVs had knowledge of where they could turn if needed compared to the control group
Stijnen et al. 2014 Netherlands [20]	Nurse‐led home visitation programme to improve health‐related quality of life and reduce disability among potentially frail community‐dwelling older people in general practice: a theory‐based process evaluation.	To examine [1] the extent to which the [G]OLD home visitation programme was implemented as planned in general practices, and [2] the extent to which general practices successfully redesigned their care delivery	24 general practices participated, of which 13 implemented the home visitation programme and 11 delivered usual care to older adults aged 75 years and older	A mixed‐methods approach, the focus is on fidelity (quality of implementation), dose delivered (completeness), dose received (exposure and satisfaction), reach (participation rate), recruitment, and context. Semi‐structured interviews with practice nurses (PNs), general practitioners (GPs), and older adults; feedback meetings with PNs; structured registration forms filled out by PNs; and narrative descriptions of the recruitment procedures and registration of inclusion and drop‐outs by members of the research team. A PHV is described as follows: a comprehensive geriatric assessment of older people's health and well‐being by the PN, tailored care and treatment plan, multidisciplinary care management, and targeted intervention Follow‐up over time (depending on type of problem and needs)	1: A home visit for conducting a comprehensive geriatric assessment, a tailored care and treatment plan, multidisciplinary care management, and targeted intervention and follow‐up. 2: Older adults were satisfied with PHV. They had a positive attitude towards the nurse and good conversations. Older adults were more likely to contact the health centre when needed after PHVs. Those who did well did not find PHVs helpful
Tøien et al. 2014 Norway [21]	How Do Older Persons Understand the Purpose and Relevance of Preventive Home Visits? A Study of Experiences after a First Visit	To explore and describe older persons' experiences of their first PHV	10 participants (75 years old)	Exploratory and descriptive design with a qualitative approach. Follow‐up: at a minimum, one annual follow‐up visit is offered, further visits may be offered if necessary. A PHV is described as follows: the purpose is to gain a comprehensive understanding of older adults' health and home situations through an open conversation. A thematic guide is applied that includes: life history, physical and mental health, functional ability, home safety and need for home modifications, activities, life style, nutrition, family, and social network	1: Information was given based on the individual's needs regarding lifestyle. 2: Many experienced PHVs as positive but those who were healthy had no need for it. The knowledge about follow‐up and having a contact person gave a sense of security. The relationship made possible conversations about important aspects. Conversations about risk factors provided preparation for the future. This was important even if there was no need for help during the visit. Information about social activities was appreciated and information about lifestyle was easy to understand. Getting help with assistive devices contributed to being able to continue with enjoyable activities
Tøien et al. 2015 Norway [22]	Older Users' Perspectives on the Benefits of Preventive Home Visits	To explore older people's perspectives on the benefits of preventive home visits (PHVs), after long‐term follow‐up	10 participants (81–91 years old)	Explorative design with a qualitative, hermeneutical approach. Follow‐up: the same person meets the user again the following year or as often as deemed necessary. A PHV is described as follows: the purpose is to gain a comprehensive understanding of older adults' health and home situations through an open conversation. A thematic guide is applied that includes: life history, physical and mental health, functional ability, home safety and need for home modifications, activities, life style, nutrition, family, and social network	1: Older adults received information about fall prevention and how homes could be secured. They received individual counselling to cope with basic activities in everyday life. They were given information on activities, advice on laws, rules and rights as well as on home care and assistive devices. Older adults received information about voluntary organisations aimed at facilitating everyday life, as well as advice on activities to keep the body in shape. They received advice on group training and help with maintaining relationships. They received a contact person. The nurse gave advice, information and help with assistive devices to enable continued participation in activities. Autonomy was preserved through information about self‐care. Simpler assistive devices could be provided by nurses and in collaboration with an occupational therapist. 2: PHVs contributed to a sense of security by allowing the older adults to prepare, through regular contact with the nurse and the relationship created with the nurse. Information about risk factors was important and support and advice gave the feeling of being prepared for the future. Information was given about assistive devices to be able to maintain relationships and make new contacts
Tøien et al. 2018 Norway [23]	An exploration of factors associated with older persons' perceptions of the benefits of and satisfaction with a preventive home visit service	To assess older persons' perceived benefits and opinions of a PHV service and explore associations between perceived benefits from PHV and relevant sociodemographic/health‐related factors	161 participants (77–96 years old)	A survey with a cross‐sectional design was administered during the spring of 2013 in a Norwegian municipality where nurses had offered annual PHVs to residents. Follow‐up: the same nurse repeated the visit to the older adult each year. A PHV is described as follows: the purpose is to gain a comprehensive understanding of older adults' health and home situations through an open conversation. A thematic guide is applied that includes life history, physical and mental health, functional ability, home safety and need for home modifications, activities, lifestyle, nutrition, family, and social network	1: Not described 2: Thirty‐nine percent of older adults reported that PHVs contributed to their sense of safety. The importance of PHVs regarding feeling of safety increased with age. The majority felt that PHVs contributed to being able to live a good life at home. Those who did not live alone experienced less support in being able to stay at home, while those with the worst health experienced the most support in this regard. Those with a high level of mental illness experienced PHVs as very significant
Tøien et al. 2020 Norway [24]	‘A longitudinal room of possibilities’ – perspectives on the benefits of long‐term preventive home visits: A qualitative study of nurses' experiences	To describe nurses' experiences of the benefits from long‐term follow‐up with annual PHVs to a general population of older people within a Norwegian context	Ten nurses delivered the service to approximately 2000 home‐dwelling seniors aged 75–97 years	A qualitative study of nurses' experiences. Follow‐up: annual visits are offered and additional visits if it deemed necessary. The service continues as long as the older adult wants it, until they need more comprehensive healthcare services, or the person dies. A PHV is described as follows: there is a thematic guide for conversation during the visits, covering life history, physical and mental health status, functional ability, nutrition, activities, the home, family, and social network. Support is offered related to individual needs and preferences within these areas	1: Advice was given on common problems such as sleep, digestion or incontinence. Information about group training, and about laws and regulations and where they could turn if needed was given to the older adults. Also, information about how the healthcare service functioned. The older adults' knowledge and awareness were increased, which contributed to health, well‐being and independence. Relationships were prioritised at the first visit in an effort to support the older adults' sense of value, self‐esteem and security. The nurses helped the older adults to overcome barriers to participating in meaningful activities and meeting social needs. They assessed the home environment and provided information on assistive devices to make it possible to stay at home and collaborated with an occupational therapist when necessary. Information on healthy lifestyles was provided. Respect for the autonomy of the older adults was fundamental. 2: A contact person led to a trusting relationship. Information about laws and rights and about the municipality's healthcare made the older adults prepared for the future. Older adults responded well to advice on good lifestyle habits. Information about social activities was appreciated and often led to changed lifestyles

The two research questions covering four categories, respectively, see Table 3.

**TABLE 3:** Visualisation of research questions and related categories.

Research questions	Categories
What are the key areas of nursing practice in PHVs?	Supporting older adults' autonomy and independence
	Creating a relationship between the older adult and the nurse
	Advising to prevent ill health and promoting health
	Providing helpful information
What are the older adults' perspectives to PHVs?	Ranging from improving well‐being to offering nothing of benefit
	Maintaining independence and autonomy
	Having a person to contact was important
	Appreciating information, advice and support

### 3.1 What Are the Key Areas of Nursing Practice in PHVs?

#### 3.1.1 Supporting Older Adults' Autonomy and Independence

The key areas of nursing practice in PHVs were described as supporting the autonomy and independence of older adults. Respect for older adults' right to determine their own lives was described as fundamental during the visit. Independence could be strengthened by the nurse during PHVs by providing knowledge and support to the older adult [24]. To enable older adults to continue living independently for longer periods, nurses could offer an assessment of their home, provide information about available assistive devices, and explore options for financial and practical assistance to adapt the home [24]. Moreover, autonomy could be maintained through information on self‐care, which could reduce their dependence on healthcare services [22].

To be able to increase autonomy and independence during PHVs, nurses could provide simple assistive devices that could be adjusted as needs changed over time [22]. For instance, they might provide a shower chair to enhance safety while bathing/showering [15]. If there was a need for more advanced or extensive assistive devices, nurses could contact physiotherapists who would conduct assessments in the home [22]. Occupational therapists could also collaborate on the need for assistive devices, not only by prescribing them but also by finding assistive devices that target older adults' needs [24].

#### 3.1.2 Creating a Relationship Between the Older Adult and the Nurse

Creating a trustful relationship with the older adult was described as important during the PHV. Nurses experienced that good relationships were promoted and that they gained the trust of the older adults [17]. Nurses conducting PHVs noted that the relationship with the older adult was enhanced by conducting visits in their homes, as the home provided a familiar and secure environment for many older adults. Prioritising the relationship during the initial visit was described by the nurses as a strategy to support the older adults' sense of worth, self‐esteem, and security [24]. If the established trust deteriorated, it could lead to the older adult becoming more reserved; hence, continuous relationship‐building during follow‐ups was deemed essential [22]. The nurse conducting the PHV then became the older adult's primary contact nurse [15, 16, 22]. Moreover, nurses reported that the same contact person allowed for a trusting relationship that enabled the older adults to talk about their problems [24].

#### 3.1.3 Advising to Prevent Ill Health and Promoting Health

The scientific literature reported that nurses often advised older adults about how to prevent ill health and promote health. Fall prevention strategies [22] and specific suggestions on how to prevent falls [17] were discussed, along with information on how older adults could avoid accidents by seeking external assistance for tasks such as shoveling snow, changing light bulbs, and hanging curtains, activities that may pose risks for older adults [15]. Nurses provided advice on common issues such as sleep, digestion, or incontinence [24]. The advice provided during the PHVs was tailored to older adults' individual needs and described as simple advice on self‐care, including maintaining a healthy and regular diet [21]. Additionally, nurses offered feedback on the older adults' current lifestyle habits concerning diet and exercise [18], and advice on how to maintain physical fitness [22]. Older adults received individual support and practical tips to manage activities of daily living, as well as advice on making their homes safer [22]. In both Germany and Sweden, nurses collaborated with other professionals, such as physiotherapists, on exercise programs for older adults, aiming to preserve physical abilities [16] and to prevent future disabilities [15]. Nurses also recommended group exercises led by municipal physiotherapists [22, 24]. Moreover, nurses advised about how the person could overcome various obstacles to participating in meaningful and enjoyable activities, thereby meeting social needs [24] and maintaining relationships [22]. Advising about social activities often resulted in positive changes in the older adult's lifestyle, according to nurses experienced in PHVs [24].

#### 3.1.4 Providing Helpful Information

Older adults received brochures containing information [15, 18]. Several studies revealed that older adults were provided with information about laws, regulations, and rights, as well as where to seek assistance if they needed home care services or assistive devices [15, 22, 24]. They also received information about various voluntary organisations that could support their daily lives. This could include assistance with grocery shopping or a meal service known as “meals on wheels” [22]. Through PHV, older adults received information about various activities available to them [16, 22]. Nurses conducting PHVs found that information about older adults' statutory rights and the municipality's various healthcare initiatives provided them for future health problems [24].

### 3.2 What Are the Older Adults' Perspectives to PHVs?

#### 3.2.1 Ranging From Improving Well‐Being to Offering Nothing of Benefit

Older adults perceived the visit as contributing to their overall well‐being, while some felt it offered nothing for them. A common perception among older adults was that PHV represented something positive and meaningful [15, 16, 18, 20, 21, 22]. The reasons for this varied, but some felt that through PHV, they became valued members of society [15], some felt appreciated [18, 19], and some described an increased self‐confidence and improved well‐being by feeling seen [16]. PHVs seem to be very valuable for older adults with mental health issues [23]. On the other hand, some older adults felt that they managed their daily lives well and were too healthy to require PHVs; thus, they often declined them [15, 19, 20, 21]. Even those who were very ill declined PHV, as these visits were perceived as demanding, and their illness or symptoms hindered participation or the ability to absorb the content. Some did not want to think about future needs as long as they could manage independently at home [15].

#### 3.2.2 Maintaining Independence and Autonomy

Older adults perceived that the visits could contribute to maintained independence and autonomy. Through the knowledge and information that they received during PHVs, they could make more informed choices, thereby promoting their independence [16]. Many older adults believed that PHVs contributed to their ability to live a good life at home [23], and they felt that PHV encouraged confidence in their resources [16]. Older adults who did not live alone perceived less support in remaining in ordinary housing compared with those living alone, and those with the poorest health perceived the most support in this regard [23].

#### 3.2.3 Having a Person to Contact Was Important

Older adults highlighted that having a person to contact was important. Moreover, a contact person could reduce the feelings of helplessness and uncertainty among older adults [16]. They felt they had good conversations with the nurse during PHVs [20, 21]. The relationship with the nurse was friendly. Older adults perceived that their relationship with the nurse was strengthened when the nurse hailed from the same locality and spoke the same dialect [16]. Moreover, trust in the nurse influenced the willingness to open up and discuss important matters [21]. The older adults developed a positive attitude towards the nurses and were more likely to contact the healthcare centre when needed after a PHV [20]. Moreover, several articles indicated that security was associated with having a contact person [15, 18, 21, 22] and knowing that there was someone to turn to when needed [15, 18, 21]. Older adults said that if they developed a relationship with the contact person and trusted them, this also contributed to a sense of security [19, 22].

#### 3.2.4 Appreciating Information, Advice and Support

Older adults valued and appreciated the information, advice, and support they received during PHVs [15, 16, 18, 21, 22]. For example, older adults appreciated information about social activities [16, 20]. Many older adults appreciated receiving a brochure during a PHV with information about where and to whom they could turn to meet future needs. They were positive about the brochure being handed out personally as it allowed them to discuss the content and clarify any questions [15, 18]. Moreover, discussing how to maintain relationships and reconnect with contacts can also be important for older adults [22]. Older adults also perceived it as important to discuss assistive devices during PHVs because these could contribute to maintaining significant and enjoyable activities or tasks [21]. Through knowledge of how to strengthen their bodies, older adults described themselves as motivated to take preventive measures [15]. Several studies also show that older adults appreciated and found meaning in discussing risk factors [15, 21, 22], as it contributed to a sense of preparedness for the future [18, 21, 22], even if there was no need for assistance during the visit [21].

## 4 Discussion

To be able to guide future practices about PHVs for older adults, a review of the scientific literature is needed. This review visualises the key areas of nursing practice in PHVs as well as older adults' perspectives on the visit. This review identifies four key areas of nursing practice within the context of the visit. These areas highlight that the nurse can support autonomy and independence throughout the visit, as well as focus on building a good relationship. The visit also provides an opportunity to offer health advice and information. Older adults describe that the visit can improve well‐being, maintain independence, and support autonomy. Moreover, they emphasise the importance of having a contact person and appreciate the information, advice, and support given during the visit. In addition to the benefits, some older adults did not find the visit particularly beneficial for them.

Having a contact person seems to be an important element of the PHVs. The findings showed that having a person to contact contributed to a sense of security for older adults. The nurses explained how they worked on creating this trustful relationship as an important part of the visit. Previous research has shown that concerning health care, continuity with a contact person can reduce mortality [25], and especially when it comes to persons with complex health issues, a nurse who coordinates the care seems to be important [26]. However, this review indicates that it is important to have a contact person also when it comes to health promotion and prevention, and not only for persons dealing with complex health issues. A contact person who can guide the older adult if needed, not only within the health care system but also in relation to preventive and promotive matters. Moreover, previous research on PHVs highlights the importance of follow‐up [6, 27]. In the present findings, follow‐up was offered in seven studies both via telephone and as a visit. A newly published study has shown that telephone follow‐up, after a PHV, was valued by older adults but could be time‐consuming for the personnel [27]. Thus, follow‐ups after the first visit seem to be important, preferably with the same contact person who conducted the first visit. This is to create a trustful relationship that can contribute to security as well as maintain health and well‐being.

Advice, support, and information seem to be important aspects of the visit, and older adults appreciate these things. The present findings showed that discussing social activities and giving information about different activities offered in the municipality was appreciated by older adults. This aligns with previous research indicating that the ability to maintain social relationships and engage in social activities plays a crucial role in supporting everyday activities at home [28, 29]. PHVs target different age groups of older adults [8] and also offer the possibility to reach persons who do not typically go to these activities and help them attend. However, the present findings showed that persons who feel in good health or persons who are very ill decline the visit or see no value in it. It could be the case that the most vulnerable older adults are not reached by the visit. Moreover, there is research showing that persons in bad health do not appreciate social activities as much as those in good health [30]. Therefore, advice and support given during PHVs must be person‐centred and based on the older adults' needs and preferences.

Older adults believe that PHVs can support independence and autonomy. The findings showed that an important element of the visit was to support independence and autonomy. Older adults also said that the visit contributed to independence through, for example, encouraging confidence in their resources. Remaining independent in older age can be valuable for many people [31, 32] and is associated with life satisfaction [32]. Research shows that with increasing age, the risk of dependence will increase, which will have an impact on the healthcare system as well as informal caregivers [33]. Therefore, to increase the possibility for older adults to stay independent as long as possible, preserve their autonomy, and remain in ordinary housing, interventions like PHVs are needed.

### 4.1 Methodological Consideration: Strength and Limitations

The strength of this review lies in its inclusion of scientific literature from 2013 to 2023 and adherence to the JBI guidelines for a scoping review. Additionally, the first author has expertise in PHVs, having conducted research in this field for over 5 years.

However, there are also limitations to the review. PHVs are not always conducted by nurses, and in some of the included articles, multiple professions are involved in the visits. Therefore, it cannot be guaranteed that a nurse is always represented in the findings. Nevertheless, it is most likely that nurses conduct the majority of PHVs, as this is the most common practice [8]. Moreover, the data selection, data extraction, and analysis were conducted by students training to become district nurses with limited experience in conducting research, but they received continuous supervision from a senior researcher, which strengthened the findings.

## 5 Conclusion

This review highlights the important role of PHVs in enhancing older adults' independence and well‐being. It lays the groundwork for future research towards guidelines about PHVs. The findings emphasize the nurse's role in not only providing assessments and coordinating preventive work but also in building trusting relationships that enhance security. Moreover, some older adults felt that the visits did not offer anything for them. This is important to be aware of and tailor the visits to the needs of older adults and those who would benefit most from the visits; for example, age may not always be the best factor to consider when determining who should be offered a visit. Therefore, in clinical practice, it is important to see the person within her unique context.

PHVs offer tailored advice for illness prevention and health promotion, covering fall prevention, physical fitness, and social engagement. By addressing individual needs, PHVs support older adults in maintaining their independence and health. Future research should focus on the key areas identified in this review to develop an effective intervention model for PHVs. Moreover, the present findings could be used in practice to include older adults' perspectives in the development of PHVs.

## Author Contributions

**Anna Nivestam:** conceptualisation, methodology, writing – original draft, writing – review and editing, visualisation, supervision. **Josefine Jöninger and Julia Jansson:** formal analysis, validation, data curation, writing – original draft, writing – review and editing. **Cecilia Pettersson:** methodology, formal analysis, validation, writing – original draft, writing – review and editing, visualisation, supervision.

## Ethics Statement

The authors have nothing to report.

## Conflicts of Interest

The authors declare no conflicts of interest.

## Data Availability

Data sharing not applicable to this article as no datasets were generated or analysed during the current study.
